# Surface disinfection procedure and in vitro regeneration of grapevine (*Vitis vinifera* L.) axillary buds

**DOI:** 10.1186/s40064-016-2081-0

**Published:** 2016-04-14

**Authors:** M. F. Lazo-Javalera, R. Troncoso-Rojas, M. E. Tiznado-Hernández, M. A. Martínez-Tellez, I. Vargas-Arispuro, M. A. Islas-Osuna, M. Rivera-Domínguez

**Affiliations:** Centro de Investigación en Alimentación y Desarrollo, A.C. Coordinación de Ciencia de los Alimentos, Carretera a La Victoria Km 0.6, 83304 Hermosillo, Sonora México; Centro de Investigación en Alimentación y Desarrollo, A.C. Coordinación de Tecnología de Alimentos de Origen Vegetal, Carretera La Victoria Km 0.6, 83304 Hermosillo, Sonora México

**Keywords:** Flame seedless, Grape, Isothiocyanate, Micropropagation, Organogenesis, Clonal propagation

## Abstract

Establishment of an efficient explants surface disinfection protocol is essential for in vitro cell and tissue culture as well as germplasm conservation, such as the case of Grapevine (*Vitis* spp.) culture. In this research, different procedures for disinfection and regeneration of field-grown grapevine cv. ‘Flame seedless’ axillary buds were evaluated. The buds were disinfected using either NaOCl or allyl, benzyl, phenyl and 2-phenylethyl isothiocyanates. Two different media for shooting and four media for rooting were tested. Shoot and root development per buds were registered. The best disinfection procedure with 90 % of tissue survival involved shaking for 60 min in a solution containing 20 % Clorox with 50 drops/L Triton^®^ X-100. These tissues showed the potential to regenerate a complete plant. Plant regeneration was conducted using full strength Murashigue and Skoog (MS) medium supplemented with 8 µM benzyl aminopurine for shoot induction and multiplication, whereas rooting was obtained on half strength MS supplemented with 2 mg L^−1^ of indole-3-butyric acid and 200 mg L^−1^ of activated charcoal. In this work, it was designed the protocols for obtaining sterile field-grown grapevine buds and in vitro plant development. This methodology showed potential to produce vigorous and healthy plants in 5 weeks for clonal grapevine propagation. Regenerated plants were successfully established in soil.

## Background

Grape (*Vitis vinifera* L.) is considered one of the most economically important crops in the world (Wang et al. 2004). Therefore, it is important to propagate this woody plant because of its commercial value in wine production, fresh consumption and juice production. In México, the greatest area dedicated to the growth of table grapes, was reported in Sonora State, with 19,870 hectares, which corresponds to 69 % of the national production (SIAP 2012). In Sonora State, the main cultivated varieties are ‘Perlette’, ‘Flame seedless’, ‘Sugraone’, and ‘Red Globe’ (AALPUM 2012).

Exploitation, biotic and abiotic stresses constantly alter grapevine crops with negative effects on quality and production levels. It is imperative to conserve these agronomic grapevine varieties and prevent the loss of plant genetic material. That is why some species are maintained in germplasm banks to keep their genetic diversity, which is necessary for plant breeding programs (Schuck et al. 2011). However, it is hard to conserve woody plants in gene banks. The genetic diversity of perennial plants, including grapevines, is usually maintained in field gene banks (Santana et al. 2008; Leão and Motoike 2011). However, these collections are constantly in danger due to exposure to the environment; therefore, the conservation of these species requires the development of efficient and cost-effective ex situ protocols, which can be complemented with in situ preservation programs (Touchell et al. 2002).

Biotechnological strategies, based on in vitro plant tissue and organ culture, have been developed to overcome these problems (Scherwinski-Pereira and Costa 2010; Vasanth and Vivier 2011). Breeding programs for species such as grapevine are time consuming because of their long life cycle (Bouquet 1989). Because of this, more than 80 % of grapevine plants have been proliferated for many centuries through vegetative propagation (Meredith 2001). Unfortunately, tissues of field-grown plants are highly contaminated. Consequently, it is difficult to obtain sterile explants suitables for in vitro tissue culture protocols (Rugini 1990).

Traditionally, the disinfection method uses chloride hypochlorite solutions (NaOCl), which usually represents a good option for tissue disinfection (Wong 2009; Norton and Skirvin 2001; Ibañez et al. 2005). However, that procedure depends on several factors, including explant source, mother plant age, cultivar and genotype (Haissig 1974; Kozlowski 1992; Friend et al. 1994; Howard 1994). In the case of field-grown plant tissues with many microorganisms from the soil and environment, it is necessary to search for alternative protocols to obtain sterile tissues to start a protocol for in vitro plant tissue culture.

Isothiocyanates (ITCs) are considered to be a promising candidate as natural antimicrobial agents. ITCs are sulfur- and nitrogen-containing secondary compounds that are characteristic of the *Brassicaceae* family and exhibit biocidal activity against various pathogens, including fungi, bacteria, insects and pests (Tiznado-Hernández and Troncoso-Rojas 2006; Báez-Flores et al. 2011; Troncoso-Rojas and Tiznado-Hernández 2007). ITCs are present in several tissues, such as seeds, stem, leaves, and roots of cruciferous plants (Okano et al. 1990; Clark 1992; Ohta et al. 1995). One of the major compounds of ITC is allyl isothiocyanate (AIT) (Matan et al. 2006). The antifungal and antibacterial capability of AIT has been shown (Troncoso et al. 2005), and it is known to interact with the sulfur of the cysteine and amine group of lysine inhibitor, which can inhibit the growth of the microorganism by causing oxidative cleavage of the disulfide bond and inactivation of the intracellular enzymes (Breier and Ziegelhöffer 2000; Kawakishi and Kaneko 1987).

One of the in vitro culture’s main goals is to obtain sterile explants for growth and development into a complete plant. A crucial requirement for the success of in vitro conservation is the development of efficient protocols for micropropagation. Several studies have been carried out for micropropagation of grapevine species, such as culture from shoot apices (Barlass and Skene 1978; Fanizza et al. 1984; Goussard 1981; Monette 1983; Morini et al. 1985) and axillary buds (Gribaudo and Fronda 1991; Jona and Webb 1978), mainly from plants grown in a greenhouse. However, a good challenge is still the utilization of tissues from field-grown plants. This is mainly due to the fact that the same disinfection techniques cannot be utilized for all species, cultivars, and tissue explants because there are differences in their susceptibility to different compounds and concentrations of disinfection solutions (Mihaljević et al. 2013). For this reason, the development of an efficient in vitro explant disinfection procedure is imperative to obtain good plant regeneration in order to start programs for crop improvement. Because of the above mentioned, the objective of this work was to obtain an efficient disinfection and regeneration procedure for in vitro propagation of grapevine buds obtained from the field.

## Methods

### Plant material

Stems of 70 cm in length of grapevine (*V. vinifera* L.) cv. ‘Flame seedless’ containing between 5 and 7 axillary buds were randomly selected from field growing plants of the vineyard ‘Casas Grandes’ located at km 40 of the Highway 36 North towards Hermosillo Coast, Sonora, México (29°02′41.0″N, 111°43′59.3″W).

### Disinfection assay with NaOCl solutions

The grape stems were disinfected with a solution containing commercial chlorine (NaOCl 1 %, v/v), and washed three times with tap water. Axillary buds were dissected with a sterile razor blade, and the first layer was excised and three treatments were tested. In Treatment 1 (T1), the dissected buds were immersed in 10 % commercial chlorine (0.6 % NaOCl) and 0.1 % Tween-20 solution (Wong 2009) with shaking at 100 rpm for 15 (T1-15), 30 (T1-30), and 60 min (T1-60) at 25 °C. In Treatment 2 (T2), dissected buds were immersed in 25 % commercial chlorine (1.3 % NaOCl) and 50 drops L^−1^ of Triton^®^ X-100 solution (Sigma) (Norton and Skirvin 2001) and shaken at 100 rpm for 15 (T2-15), 30 (T2-30) and 60 (T2-60) min at 25 °C. In Treatment 3 (T3), stems were pre-treated with a benomyl (100 ppm) solution for 3 min, and washed with an excess of sterile distilled water. After this treatment, axillary buds were dissected with a sterile razor blade and the first layer of the buds were excised, dipped in 70 % ethanol for 30 s, and transferred to 20 % commercial chlorine (1 % NaOCl) and a 0.1 % Tween-20 solution (Ibañez et al. 2005) with shaking at 100 rpm for 15 (T3-15), 30 (T3-30) and 60 (T3-60) min at 25 °C. Buds treated with sterile distilled water were used as controls. After the treatments mentioned, buds were rinsed three times with sterile, distilled water and placed in full strength Murashige and Skoog (MS) medium (Murashige and Skoog 1962) and incubated in a growth room at 26 °C under 12 h in photoperiod provided by white fluorescent tubes with an intensity between 32 and 40 μmol m^−2^ s^−1^. Three replicates of 36 buds were used for each treatment. The percentage of contamination was calculated by visual evaluation of bacteria or fungi presence in the buds and registered every day.

### Disinfection assay with isothiocyanate solutions

Axillary buds were dissected with a sterile razor blade and the first layer was excised. Five ITC treatments were tested, described next. Allyl isothiocyanate (A), benzyl isothiocyanate (B), phenyl isothiocyanate (P), 2-phenylethyl isothiocyanate (PE) and the mixture (Mix) of Allyl:Benzyl:2-Phenylethyl:Phenyl in a proportion of 1:3.5:5.3:9.6 (v/v/v/v) were used. All treatments were tested at 0.5, 1 and 2.5 mM (Smith and Kirkegaard 2002; Troncoso et al. 2005). For the ITC treatments, the dissected buds were placed on Petri dishes with a sterile filter paper (2 cm diameter) that was soaked with the appropriated volume of the different isothiocyanate solution and adhered to the cover of the Petri dish. After that, the dish was sealed with a strip of polyethylene film and incubated for 6 and 12 h at 28 °C. As a control, paper soaked with sterile distilled water was used. Additionally, the best procedure obtained in the first assay (T2-60: commercial chlorine at 25 % (1.3 % NaOCl) and 50 drops L^−1^ Triton^®^ X-100 detergent solution with 60 min of incubation) was used as a second control. After the incubation time, the buds were placed in full strength MS medium and maintained in a growth room at 26 °C under 12 h in photoperiod provided by white fluorescent tubes with an intensity between 32 and 40 μmol m^−2^ s^−1^. Three replicates of 36 buds were utilized for each treatment. The percentage of contamination was calculated with visual evaluation of presence of fungi or bacteria in the buds and registered every day.

### Shoot proliferation

The potential of the buds without contamination for plant regeneration from the disinfection assays were evaluated firstable for shoot proliferation. Two shoot-inducing medium were tested (SM1 and SM2). SM1 is a modified medium reported by Jaskani et al. (2008) containing full strength MS basal mineral and vitamin medium supplemented with 8 µM 6-benzyl amino purine (BAP). By other side, SM2 is a media reported by Abido et al. (2013) consisting in full strength MS medium supplemented with 1 mg L^−1^ BAP and 0.1 mg L^−1^ naphthalene acetic acid (NAA). The number of regenerated buds and shoot development per bud were visually evaluated.

### Root proliferation

Shoots obtained from the shoot proliferation medium with about 2 cm in length, it was cut at the base and placed in rooting medium. Four rooting medium were tested. RM1 reported Jaskani et al. (2008) containing full strength MS salts and vitamins supplemented with 2.5 µM indole-3-butyric acid (IBA), RM2 reported by Norton and Skirvin (2001) containing full strength MS salts and vitamins without hormone, RM3 reported by Hamidullah et al. (2013) containing half strength MS salts and vitamins supplemented with 2 mg L^−1^ IBA and 200 mg L^−1^ of activated charcoal (AC), and RM4 containing half strength MS salts and vitamins supplemented with 2 mg L^−1^ IBA and 3 g L^−1^ AC. The time and percentage of shoots developing roots were visually evaluated.

All the media were supplemented with 3 % (w/v) sucrose and it was adjusted to pH 5.8 ± 1 before the addition of 0.6 % (w/v) Gelrite^®^ gellan gum (Sigma Aldrich. St. Louis, MO, 63178, USA), and then autoclaved at 121 °C and 1.05 kg cm^−2^ for 15 min. The cultures were kept in a growth room at 26 °C under 12 h of photoperiod provided by white fluorescent tubes with intensity between 32 and 40 μmol m^−2^ s^−1^. Ten replicates consisting in three buds per flask with 20 mL of culture medium were used.

### Statistical evaluation

The data recorded were expressed as a percentage. Data generated in all the experiments was statistically analyzed by a completely random design with a significance level of 5 %. Comparison between treatments was carried out by one-way analysis of variance and the differences between the means were tested using the Tukey–Kramer multiple mean comparison procedure when the variance analysis was significant. Data homogeneity was evaluated with the Kruskal–Wallis test. All data were analyzed using NCSS statistical package (NCSS, Kaysville, Utah, USA) version 2007.

## Results

### Disinfection assay with the NaOCl solutions

**Fig. 1:**
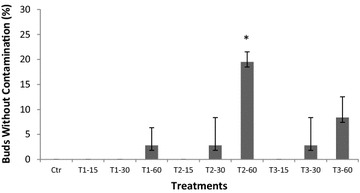
Percentage of grapevine buds of cv. ‘Flame seedless’ without contamination exposed to different NaOCl treatments on Murashige and Skoog medium at 26 °C. Ctr: buds washed with sterile distilled water T1-15, T1-30 and T1-60: buds treated with NaOCl (0.6 %) + Tween-20 incubated for 15, 30 and 60 min, respectively. T2-15, T2-30 and T2-60 buds treated with NaOCl (1.3 %) + Triton^®^ X-100 incubated for 15, 30 and 60 min, respectively. T3-15, T3-30 and T3-60 buds pre-treated with benomyl (100 ppm) and 70 % ethanol for 3 min, and then in NaOCl (1 %) + Tween-20 solution incubated for 15, 30 and 60 min, respectively. Data represent the mean of 12 buds per treatment replicated three times. *Asterisk* indicates differences among the treatments according to Tukey–Kramer test at P < 0.05

The best treatment for tissue disinfection was T2-60 with 19.8 % of buds without contamination, and it was statistically significant compared with the control (P < 0.05). For the other treatments, the percentages of buds without contamination were 1, 4 and 8 % for T1, T3 and the other T2 treatments, respectively (Fig. 1). Additionally, the employment of chlorine solutions and Tween-20 in T1 and T3 treatments, respectively, did not reduced contamination. Finally, the chlorine solution and Triton^®^ X-100 in incubation with shaking for 60 min (T2-60) was determined to be the most effective treatment for surface disinfection of grapevine field-grown buds (Fig. 1). These results also showed that the increase in the shaking time in all treatments reduced the contamination percentage.

### Disinfection assay with isothiocyanate solutions

**Fig. 2:**
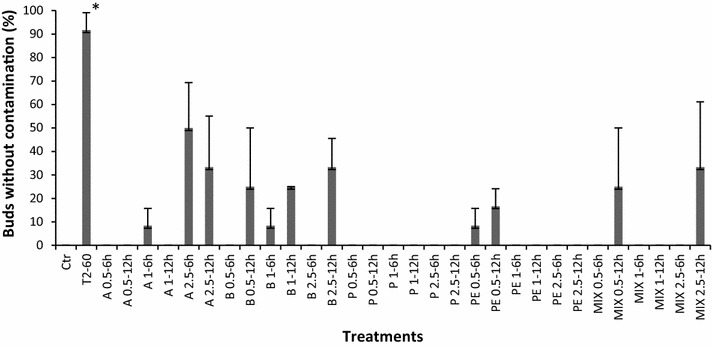
The percentage of grapevine buds cv. ‘Flame seedless’ without contamination exposed to different isothiocyanate disinfection treatments on Murashige and Skoog medium at 26 °C. Ctr: buds washed with sterile distilled water, T2-60 (second control): buds treated with NaOCl (1.3 %) + Triton^®^ X-100 incubated by 60 min. Buds treated with isothiocyanate solutions, A0.5-6 h: 0.5 mM Allyl incubated by 6 h, A0.5-12 h: 0.5 mM Allyl incubated by 12 h, A1-6 h: 1 mM Allyl incubated by 6 h, A1-12 h: 1 mM Allyl incubated by 12 h, A2.5-6 h: 2.5 mM Allyl incubated by 6 h, A2.5-12 h: 2.5 mM Allyl incubated by 12 h, B0.5-6 h: 0.5 mM Benzyl incubated by 6 h, B0.5-12 h: 0.5 mM Benzyl incubated by 12 h, B1-6 h: 1 mM Benzyl incubated by 6 h, B1-12 h: 1 mM Benzyl incubated by 12 h, B2.5-6 h: 2.5 mM Benzyl incubated by 6 h, B2.5-12 h: 2.5 mM Benzyl incubated by 12 h, P0.5-6 h: 0.5 mM Phenyl incubated by 6 h, P0.5-12 h: 0.5 mM Phenyl incubated by 12 h, P1-6 h: 1 mM Phenyl incubated by 6 h, P1-12 h: 1 mM Phenyl incubated by 12 h, P2.5-6 h: 2.5 mM Phenyl incubated by 6 h, P2.5-12 h: 2.5 mM Phenyl incubated by 12 h, PE0.5-6 h: 0.5 mM Phenylethyl incubated by 6 h, PE0.5-12 h: 0.5 mM Phenylethyl incubated by 12 h, PE1-6 h: 1 mM Phenylethyl incubated by 6 h, PE1-12 h: 1 mM Phenylethyl incubated by 12 h, PE2.5-6 h: 2.5 mM Phenylethyl incubated by 6 h, PE2.5-12 h: 2.5 mM Phenylethyl incubated by 12 h, Mix0.5-6 h: 0.5 mM Allyl:Benzyl:Pheniyethyl:Phenyl (1:3.5:5.3:9.6) incubated by 6 h, Mix0.5-12 h: 0.5 mM Allyl:Benzyl:Pheniyethyl:Phenyl (1:3.5:5.3:9.6) incubated by 12 h, Mix1-6 h: 1 mM Allyl:Benzyl:Pheniyethyl:Phenyl (1:3.5:5.3:9.6) incubated by 6 h, Mix1-12 h: 1 mM Allyl:Benzyl:Pheniyethyl:Phenyl (1:3.5:5.3:9.6) incubated by 12 h, Mix2.5-6 h: 2.5 mM Allyl:Benzyl:Pheniyethyl:Phenyl (1:3.5:5.3:9.6) incubated by 6 h, Mix2.5-12 h: 2.5 mM Allyl:Benzyl:Pheniyethyl:Phenyl (1:3.5:5.3:9.6) incubated by 12 h. All treatments were maintained at 28 °C. Data represent the mean of 12 buds per treatment replicated three times. *Asterisk* indicates differences among the treatments according to Tukey–Kramer test at P < 0.05

The best results obtained with the isothiocyanate treatments fell into the Allyl isothiocyanate 2.5 mM, incubated during 6 h (A2.5-6 h) with 50 % of buds without contamination (Fig. 2). T2-60 (second control) was better than any of the isothiocyanate treatments showing 90 % without contamination and no tissue damage. The tissues treated with T2-60 showed the potential to regenerate, in contrast with the tissues treated with the best isothiocyanate treatment A2.5-6 h, which failed to regenerate. All ITC compounds used in this assay showed a certain level of tissue phytotoxicity based on the presence of bud browning with no regeneration.

### Shoot proliferation

**Fig. 3:**
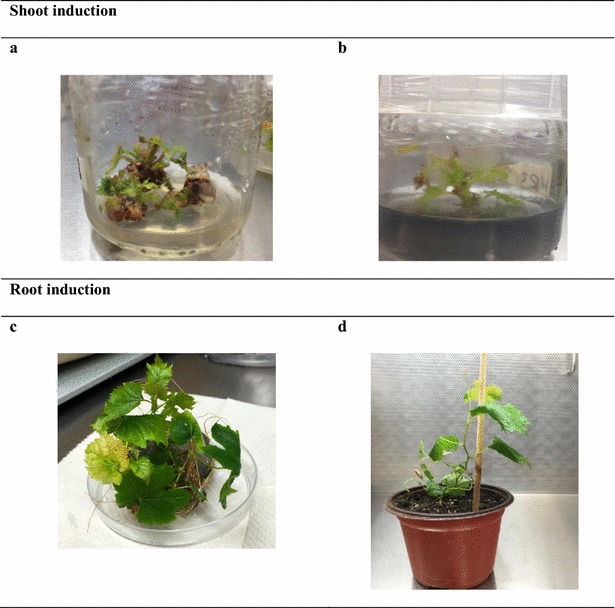
Various stages of in vitro propagation of grapevine buds cv. ‘Flame seedless’ after surface sterilization using T2-60 treatment (1.3 % NaOCl and 50 drops L^−1^ Triton^®^ X-100 solution with 60 min of incubation). **a** Multiple shoots formed from buds explant on SM1 medium (MS with 8 µM BAP), **b** shoot growing on rooting medium: RM3 (MS with 2 mg L^−1^ IBA + 200 mg L^−1^ activated charcoal), **c** full plant after root induction on medium (RM3) during 3 weeks, and **d** established plant in soil after 5 weeks of culturing

The media, SM1 and SM2 were chosen to compare the effect on shoot multiplication. Shoot multiplication in both media were recorded, but there was a significant increase (P < 0.05) in the SM1 medium (8 µM BAP) compared with the SM2. Furthermore, the development of one to three shoots per bud in SM2 medium and five to eight shoots per bud in SM1 medium was observed. Very few axillary buds (20 %) were able to sprout and grow in SM2 medium, suggesting that the NAA hormone showed less effect on shoot proliferation in grapevine buds. Among the growth regulators tested, BAP (8 µM) in SM1 was the most effective to induce shoots with a maximum of 67 % of buds that were able to developing many shoots (Table 1). The development of shoots was scored around 2 weeks after treatment initiation (Table 1; Fig. 3).

**Table 1:** Effects of different medium conditions in shoot and root proliferation on grapevine (*V. vinifera* L.) buds cv. ‘Flame seedless’

Medium	Buds producing shoots (%) (mean ± SD)	Time of shoot induction (weeks)	Number of shoots per bud (mean ± SD)	Time of root induction (weeks)	Percentage of shoots showing roots (mean ± SD)
Shooting medium					
SM1	67 ± 1.5^a^	2	8 ± 2^a^	–	–
SM2	20 ± 5.6	2	3 ± 1	–	–
Rooting medium					
RM1	–	–	–	8	0
RM2	–	–	–	8	0
RM3	–	–	–	3	62^a^
RM4	–	–	–	6	20

SM1, full strength MS with 8 µM 6-benzyl amino purine; SM2, full strength MS with 1 mg L^−1^ 6-benzyl amino purine and 0.1 mg L^−1^ naphthalene acetic acid; RM1, full strength MS with 2.5 µM indole-3-butyric acid; RM2, full strength MS without hormone; RM3, half strength MS with 2 mg L^−1^ IBA and 200 mg L^−1^ activated charcoal; RM4, half strength MS with 2 mg L^−1^ indole-3-butyric acid and 3 g L^−1^ activated charcoal. Data are the mean of 12 buds per treatment replicated three times

^a^Differences among the treatments in a column according to Tukey–Kramer test (P < 0.05)

### Root proliferation

After 8 weeks of inoculation on rooting medium, there were roots in the media without activated charcoal, and in MS full strength concentration (RM1 and RM2). The root development was obtained in the RM3 medium containing half strength MS medium with 2 mg L^−1^ IBA and 200 mg L^−1^ of activated charcoal. In this medium, 62 % of rooted plantlets were obtained after 3 weeks in culture (Table 1), reaching at least 2 cm in length. The RM4 medium was less effective at producing roots than RM3 with only 20 % of rooted shoots after 6 weeks of growth.

## Discussion

Contamination control is the key factor to success during in vitro plant tissue culture protocols. The best result with 80 % of uncontaminated buds was obtained with the T2-60 treatment because explants were undamaged and showed potential to regenerate during the shoot and root-inducing steps. Khan et al. (2002) tested four disinfection procedures in meristems of olive cv. ‘Pantaloon’ using the following conditions, P1: 15 min Clorox 100 %, P2: 70 % Ethanol + 10 min Clorox 100 %, P3: 25 min HgCl_2_ + 30 drops/L Tween-80 with 3 min + commercial bleach 50 % and P4:P3 treatment without Tween-20. P1 showed no negative effects on the tissues; however, after 3 days, 100 % contamination was recorded. Further, P3 was the best protocol. The authors concluded that the use of sodium hypochlorite as the only disinfectant was insufficient to achieve sterilization.

In the present work, using Tween-20 in the T1 and T3 treatments was not a good disinfectant due to high contamination observed at day seven (from 91 to 100 %). In contrast, Khan et al. (2002) found a synergistic effect when testing the Tween-60 and chloride ions. However, our best results were found with the utilization of Triton^®^ X-100 and Clorox at 25 % (1.3 % NaOCl). In agreement, several experiments reported good results with the utilization of NaOCl solutions (Trepagnier et al. 1977; Thé 1979; Cunningham and Balekjian 1980; Gordon et al. 1981). In the present experiment, the best results were obtained using the highest concentration of sodium hypochlorite (T2 treatment). It is well known that NaOCl has a broad-spectrum of antimicrobial activity and it is able to rapidly kill vegetative spores, bacteria, fungi, protozoa and viruses (Dychdala 1991). NaOCl exerts its antibacterial effect by inducing the irreversible oxidation of sulfhydryl groups of essential enzymes, and it may also have deleterious effects on DNA and membrane-associated enzyme activity (McDonnell and Russell 1999). Additionally, it was found that an increase in the shaking time is related to a concomitant reduction in the percentage of contamination. Grapevine buds are composed of several tissue layers, where the meristematic part is well protected. Therefore, it is not surprising that the incubation time of 60 min was optimal for all treatments, probably because it allows the penetration of the solution throughout the different tissue layers of the bud. We recorded mainly the presence of fungal contamination in the buds (data not shown) in agreement with Khan et al. (2002) that reported that the main contamination was due to fungi growth. Further, they observed the appearance of contamination after 7 days, whereas in our experiment, fungi growth was observed after 2 days.

In the case of the isothiocyanate assay, we found that the best treatment consisted of A2.5-6 h, with 50 % of no contamination (Fig. 2). Commonly, fungi have been controlled by post-harvest fungicide treatments, including thiabendazole and benomyl (Hardenburg and Spalding 1972; Tepper and Yoder 1982; Tiznado-Hernández and Troncoso-Rojas 2006). However, the presence of strains resistant to these compounds reduced the effectiveness of such treatments. For that reason, new plant tissue disinfection alternatives are needed. The treatments to control fungi infection by isothiocyanates had been tested in vitro, in field, and with harvested fruits. However, the use of these compounds in protocols to control fungal infection during plant tissue culture has not been studied. The mechanisms by which ITCs inhibit microorganism and fungal growth are not well known. Some hypotheses propose that ITCs cause inactivation of intracellular enzymes by oxidative breakdown of disulfide bridges and inhibition of metabolic enzymes by reaction with the sulfur and amine groups of the amino acids. Apparently, the high reactivity of ITC is due mostly to the strong electrophilic nature of the NCS group (Kroll et al. 1994). Experiments in vitro demonstrated that allyl-isothiocyanate could form covalent bonds with the disulfide bonds of the oxidized glutathione (Kawakishi and Kaneko 1987), as well as amino acids (Cejpek et al. 2000), suggesting that the ITCs can chemically react with almost any protein.

The best treatment with ITCs was at the level of 2.5 mM incubated for 6 h, which resulted in 50 % of buds with no contamination. However, these tissues failed to regenerate. All of the different ITCs compounds used in this study showed tissue phytotoxicity based on the presence of bud browning and the lack of bud tissue development. According to these results, these compounds cannot be used in the disinfection method in the micropropagation of grapevine buds; however we believe that it is necessary further research to probe other compounds and/or different concentrations of ITC. By the way, few reports had been available in this context. However, in agreement with the results of this investigation, it was reported that the administration of a high dose of ITC inhibited plant growth and induced severe bleaching in the rosette leaves (Hara et al. 2010).

For bud regeneration, it is important to establish the optimal composition medium for each cultivar. Previously, many studies have indicated that the ideal composition of grapevine culture medium depends on the species and cultivars, so the results obtained with one genotype in a given medium may differ from those obtained with other genotypes (Reisch 1986; Botti et al. 1993). Our results showed that the concentration of BAP was critical for stimulating explant growth and development. Shoot development from buds obtained from grapevine buds grown in the field was achieved. The presence of BAP in the culture medium was a key factor for inducing bud development.

Two media, SM1 and SM2, were chosen to compare the effect on shoot multiplication. The results showed successful induction of shoot multiplication in both media, but there was a significant increase in the SM1 medium (8 µM BAP) (Fig. 3). This response was evaluated by the number of buds regenerated and the number of shoots per bud obtained. Bernd et al. (2007) evaluated grapevine hybrids and obtained an average of 4.9 shoots per explant in a culture medium supplemented with 3.0 μmol L^−1^ of BAP. In another study with a different grapevine hybrid, multi-budding was observed at a concentration of 4.4 μmol L^−1^ of BAP (Torregrosa and Bouquet 1995). In agreement with our data, Skiada et al. (2009) reported optimum BAP concentrations between 4.0 and 16 μmol L^−1^ for in vitro propagation of *Vitis* spp. genotypes. The effect of growth regulators on explant development was clearly positive due to the medium containing a higher concentration of BAP (8 µM), which was the most effective for bud regeneration with 67 % of buds developing shoots (Table 1).

The induction of bud development is usually carried out using BAP because the inclusion of cytokinins in the medium is necessary for shoot proliferation from shoot tips and axillary buds (Goussard 1981; Torregrosa and Bouquet 1995; Gray and Fisher 1985; Lee and Wetzstein 1990; Mhatre et al. 2000). In agreement, the development of shoots from buds was observed in cultivars of *Vitis*, with a culture medium supplemented with 4.0 µM L^−1^ of BAP (Skiada et al. 2009). Furthermore, Alizadeh and Singh (2009) also reported multiple shoots in the in vitro culture of grapevines, with an optimum BAP concentration of 2.0 mg L^−1^.

Shoots obtained using SM1 and SM2 medium were placed in rooting medium. The best medium consisted of half strength MS salts, AC and 2 mg L^−1^ IBA. In agreement with our results, Lee and Wetzstein (1990) showed that in ‘Muscadine’ grape, the percentage of rooting was greater in the treatments including IBA compared with that without hormones, although this behavior was genotype dependent. Bernd et al. (2007) utilized a medium supplemented with NAA at a concentration of 8.05 × 10^−3^ μmol L^−1^ to induce rooting of grapevine shoots induced by the utilization of BAP at concentrations of 3.5 and 10 μmol L^−1^. Silva et al. (2012) obtained the highest number of roots on the grapevine at a concentration of 0.4 μmol L^−1^ of IBA, with an average of 3.3 roots per explant. Further, an average of 3.0 and 2.6 roots per explant were obtained by using 2.4 and 4.9 μmol L^−1^ IBA concentrations, respectively. It appears that rhizogenesis in grapevines seems to be strongly genotype-dependent (Lewandowski 1991). Further studies are needed to find the optimal medium for rooting for the ‘Flame seedless’ cultivar. Rooting is a key to establish a regeneration system in plant tissue culture because the development of physiologically active roots could be decisive for carrying out the transference of plants growing in vitro to the field.

The initiation of adventitious root formation can be difficult because it is regulated by internal factors, including plant growth regulators such as auxins and cytokinins, as well as the genetic background (Haissig 1974; Kozlowski 1992; Friend et al. 1994; Howard 1994). However, the role of auxin on adventitious root is still not well understood. Woody plants, such as grapevine, represent a highly complex system in which endogenous hormone levels, transport, dormancy, storage, and inhibitory compounds influence adventitious root growth, and each of these are dependent on preconditioning treatments (Andersen 1986; Wilson 1994). Spiegel (1955) argued that much of the inconsistency in adventitious root research encountered for *Vitis* tissues was, in part, due to the existence of compounds that inhibit adventitious root formation. Singh et al. (2004) reported that when actived charcoal was added with IBA, there was an increase in rooting percentage. The results obtained in our work for rhizogenesis time were comparable to those reported in many articles, suggesting that this phenomenon requires between 12 and 30 days (Reisch 1986; Bernd et al. 2007; Salami et al. 2005; Diab et al. 2011).

The protocol developed in the present study can be considered to be very good for disinfection and in vitro micropropagation of field-grown axillary buds of grapevine cv. ‘Flame seedless’. These findings could open up new prospects for grapevine clonal propagation as well as for the creation of an ex situ germplasm bank of *V. vinifera* L.

## Conclusions

It was possible to develop a fast and efficient method for disinfection for grapevine cv. ‘Flame seedless’ axillary buds obtained from field-grown. The best disinfection procedure was using a solution containing Clorox and 50 drops/L Triton^®^ X-100. With this treatment, the tissues showed the potential to regenerate in Murashigue and Skoog medium supplemented with benzyl aminopurine for shoot induction and multiplication, whereas rooting was obtained with indole-3-butyric acid and activated charcoal. Isothiocyanate compounds were found to be toxic for the tissues and no regeneration was recorded from explants treated. The protocol here developed can produce in 5 weeks healthy and vigorous plants, which can be transferred to soil for clonal grapevine propagation.
